# Hemodynamic study of the ICA aneurysm evolution to attain the cerebral aneurysm rupture risk

**DOI:** 10.1038/s41598-024-59242-w

**Published:** 2024-04-18

**Authors:** Huaying Huo, Yigang Chang

**Affiliations:** Shanxi Provincial People’s Hospital, TaiYuan, Shanxi 030012 China

**Keywords:** Wall shear stress, Aneurysm evolutions, Bloodstream, Micro cerebral, Non-newtonian flow, Biomedical engineering, Mechanical engineering

## Abstract

The influence of the aneurysm evolution on the hemodynamic characteristic of the blood flow inside the sac region is comprehensively investigated. By using the computational method, the blood flow through the vessel and aneurysm of the sac region is examined to find the role of aneurysm evolution on the wall shear stress, pressure, and risk of aneurysm rupture. Three different models of ICA aneurysms are chosen for the investigation of the aneurysm evolution at risk of rupture. Obtained data shows that the evolution of the aneurysm decreases the wall shear stress and pressure on the sac surface while an oscillatory index of blood increases on the aneurysm wall.

## Introduction

Cerebral aneurysms are abnormal bulges or weak spots in the blood vessels of the brain. These aneurysms can undergo various changes over time, leading to alterations in their hemodynamics, or blood flow patterns^1–3^. Hemodynamic changes play a significant role in the evolution of cerebral aneurysms and can affect the risk of rupture. When an aneurysm forms, it often starts as a small, localized dilation in a blood vessel. As the aneurysm grows in size, it can undergo structural and hemodynamic changes. The shape, size, and orientation of the aneurysm can influence the blood flow patterns within it. Hemodynamic changes within the aneurysm can be categorized into two main types: steady-state and transient flow changes. Steady-state flow changes occur as the aneurysm enlarges. The increased size leads to a larger volume of blood within the aneurysm, which can result in altered flow velocities and wall shear stress. These changes can impact the integrity of the aneurysm wall, potentially increasing the risk of rupture^4,5^.

Transient flow changes, on the other hand, occur due to fluctuations in the surrounding hemodynamics. Factors such as heart rate, blood pressure, and blood flow volume can vary, leading to changes in the flow patterns within the aneurysm. These transient flow changes can induce complex flow patterns, including vortex formation, flow recirculation, and high wall shear stress. These flow disturbances can contribute to the weakening of the aneurysm wall, making it more susceptible to rupture^6,7^.

The size of the aneurysm also plays a crucial role in its hemodynamic changes and rupture risk. As the size of an aneurysm increases, the hemodynamic forces acting on its walls become more pronounced. Larger aneurysms tend to have a higher risk of rupture due to increased wall tension and stress. Additionally, larger aneurysms often exhibit more complex flow patterns, further increasing the risk^8,9^.

Understanding the hemodynamic changes that occur with the evolution of cerebral aneurysms and their effects on rupture risk is vital for effective management and treatment decisions^10–12^. Medical professionals use various imaging and computational techniques to assess aneurysm characteristics and evaluate the associated hemodynamic changes. This information helps guide treatment strategies, including surgical intervention or endovascular procedures, to mitigate the risk of rupture and improve patient outcomes^13,14^.

Computational Fluid Dynamics (CFD) is a powerful tool used in the estimation and analysis of hemodynamic factors during the evolution of cerebral aneurysms^15–17^. CFD involves the mathematical modeling and simulation of fluid flow to analyze the complex blood flow patterns within the blood vessels^18–20^. In the context of cerebral aneurysms, CFD can provide valuable insights into the hemodynamic changes that occur within the aneurysm and its surrounding blood vessels. By creating a virtual model based on medical imaging data, CFD enables the simulation of blood flow and the estimation of various hemodynamic factors^21,22^.

One of the primary hemodynamic factors of interest is wall shear stress (WSS), which represents the frictional force exerted by blood flow on the vessel walls. High WSS has been associated with aneurysm growth and rupture^23–25^. CFD simulations can help estimate WSS distribution within the aneurysm and identify regions of elevated WSS that may indicate potential rupture risk. CFD also enables the visualization of flow patterns, including vortex formation, flow recirculation, and complex flow structures within the aneurysm. These flow patterns can be indicative of potential rupture sites or regions of increased wall stress. By examining these patterns, clinicians can gain a better understanding of the aneurysm’s behavior and assess its risk^26^.

Furthermore, CFD can assist in evaluating the effectiveness of different treatment strategies. By simulating blood flow before and after interventions such as stent placement or flow diversion devices, CFD can help assess their impact on hemodynamics. This information aids in decision-making regarding the optimal treatment approach for individual patients. It’s important to note that while CFD provides valuable insights, it is still a modeling approach and has limitations^27^. Assumptions and simplifications are made during the simulation process, and accuracy depends on the quality of input data and model parameters. Therefore, CFD results should be interpreted with caution and used in conjunction with clinical judgment and other diagnostic tools. In summary, CFD is a valuable tool for estimating hemodynamic factors in the process of cerebral aneurysm evolution. It enables the estimation of wall shear stress, visualization of flow patterns, and assessment of treatment strategies. Integrating CFD into clinical practice can enhance the understanding of aneurysm behavior and aid in decision-making for patient management.

Although there are several investigations in which aneurysm rupture risk is presented in different sizes and locations, the risk of rupture of the aneurysm in the evolution process is not fully examined in the available resources and articles. The main concept of this work is to perform a numerical simulation of bloodstream in the cerebral ICA aneurysms in different scales to find the risk of aneurysm rupture in the process of the aneurysm evolution. Three ICA aneurysms in different scales have been investigated in this paper at the same conditions to analyze the hemodynamic ’factors of wall shear stress and OSI in the growing process.

## Governing equations and applied computational method

It is confirming that all methods were carried out in accordance with relevant guidelines and regulations. Besides, all experimental protocols were approved by of the Emory University and it is confirmed that informed consent was obtained from all subjects and/or their legal guardian(s)^28^.

The simulation of the non-Newtonian transient bloodstream along the artery and aneurysm is done by solving the Navier–stokes equations. In the available articles, the main challenge for modeling the blood is viscosity calculation due to non-Newtonian characteristics of the blood in the vessels. Casson model is widely applied by researchers who have performed computational approaches to obtain hemodynamic results^29^. Although there are several models for the viscosity calculation of blood flow, blood hematocrit value is defined in this correlation as an input^30^. Thus, the patient’s gender is also applied in this methodology. One-way Fluid–solid interaction is also considered in this study since the vessel is deformed by the blood hemodynamic factors^31^.

**Figure 1:**
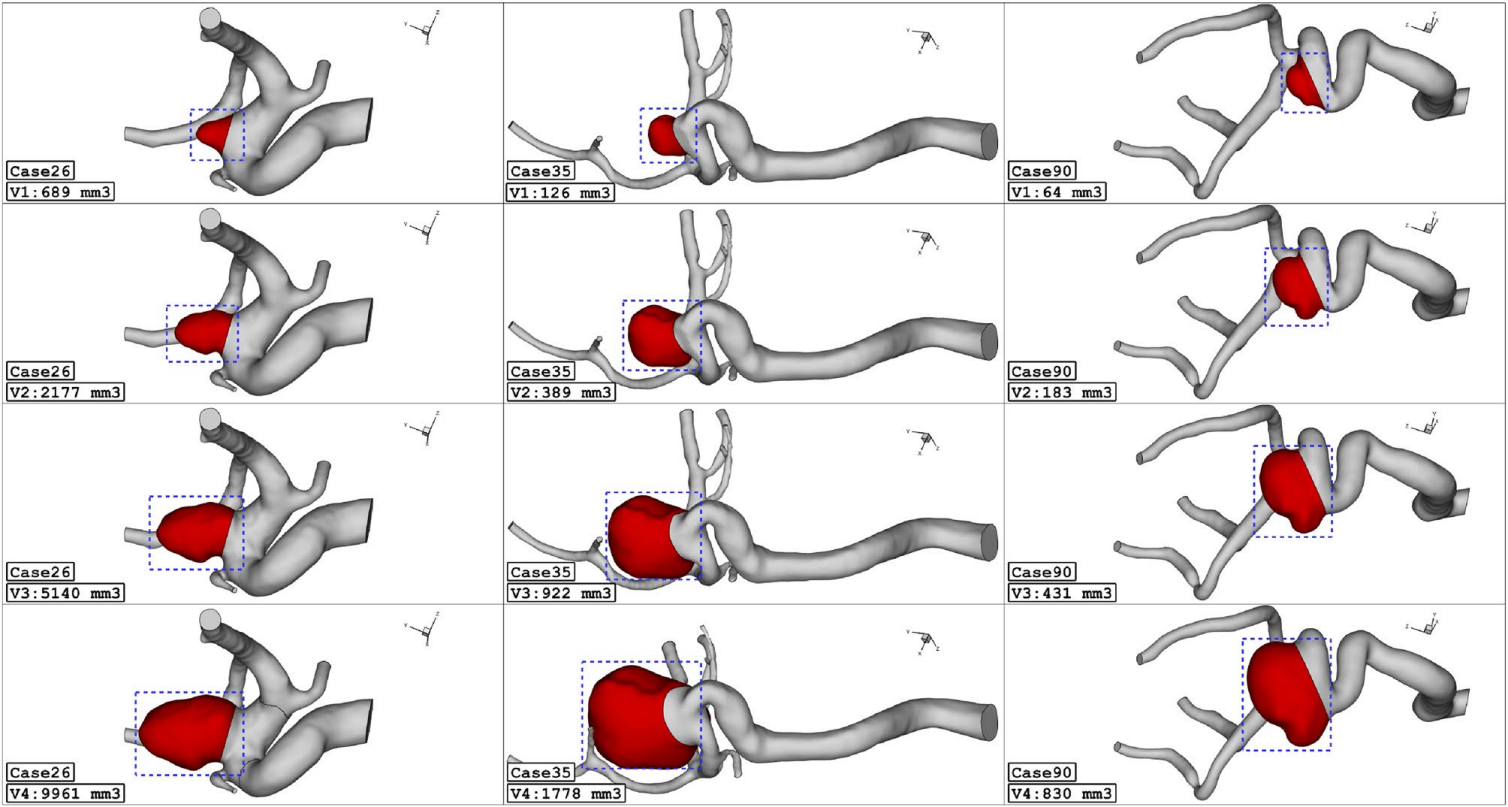
ICA aneurysm geometry of 3 different cases (Four different aneurysm volume).

**Figure 2:**
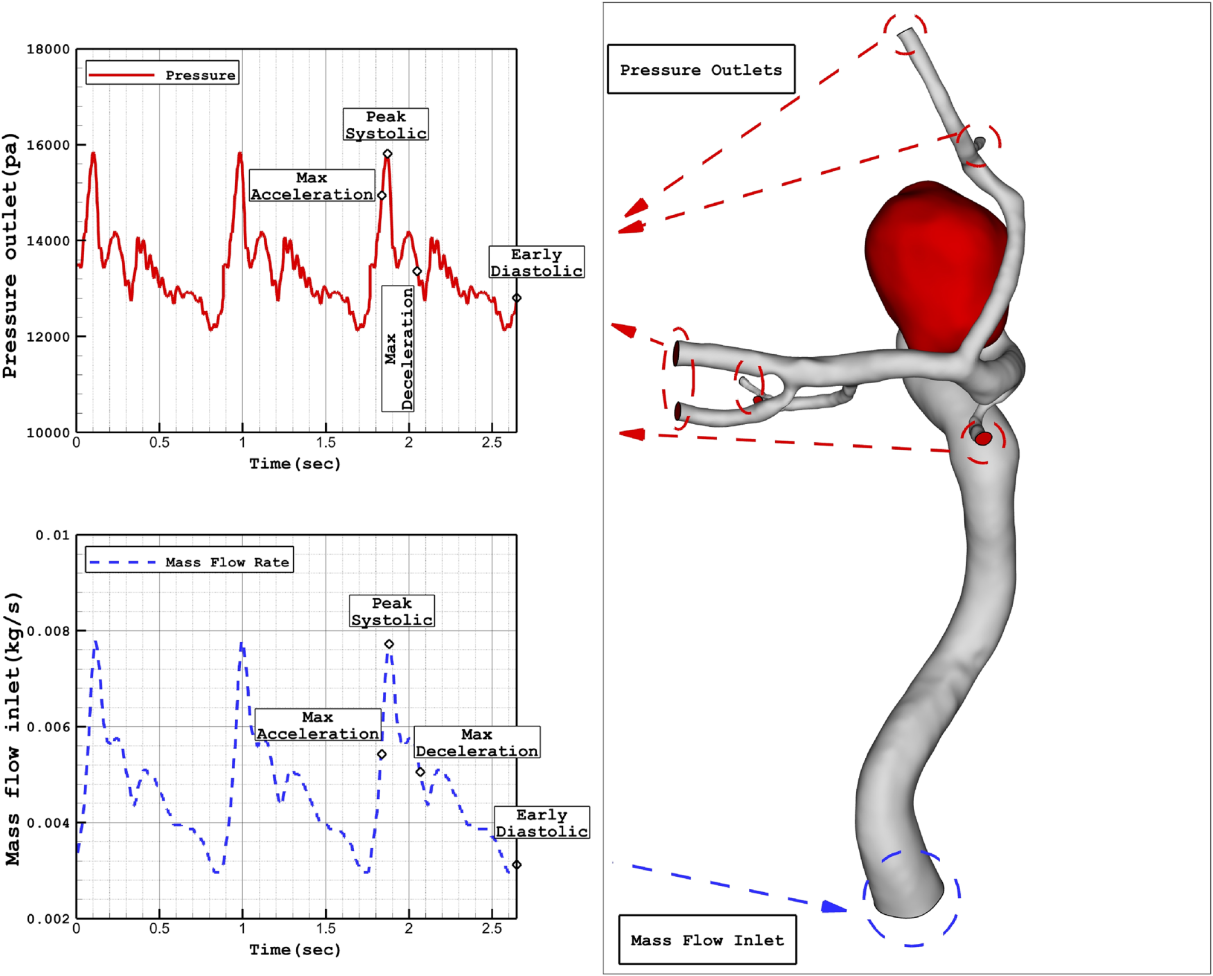
Applied profile at inlet (mass flow) and outlets (pressure outlet).

Figure 1 illustrates the geometry of the three ICA aneurysms with different sac volume sizes in three different scales. The volume of these selected aneurysms is scaled up and down to find the risk of aneurysm rupture in these volumetric conditions. The geometrical aspects of the original and scaled aneurysms are presented in Table 1. All patients are female and the blood hematocrit is 0.4 for all cases. The applied cardiac cycle on the inlet and outlet of the chosen model is schematically displayed in Fig. 2. The Mass flow rate with the introduced flow rate is applied at the inlet while the pressure outlet is used for the outlet condition of the model. The reported OSI value is calculated at the end of the third cardiac cycle. Besides, the wall shear stress is analyzed at peak systolic in which blood mass flow is maximum.

**Table 1:** Geometrical details of selected cerebral aneurysm.

Case ID	Sac Volume (mm^3^)				Sex
	Case I	Case II	Case III (Original)	Case IV	
26	688.89	2176.62	5139.47	9961.07	Female (HCT = 0.40)
35	126.45	388.68	921.91	1777.63	Female (HCT = 0.40)
90	64.15	182.56	430.51	829.65	Female (HCT = 0.40)

**Figure 3:**
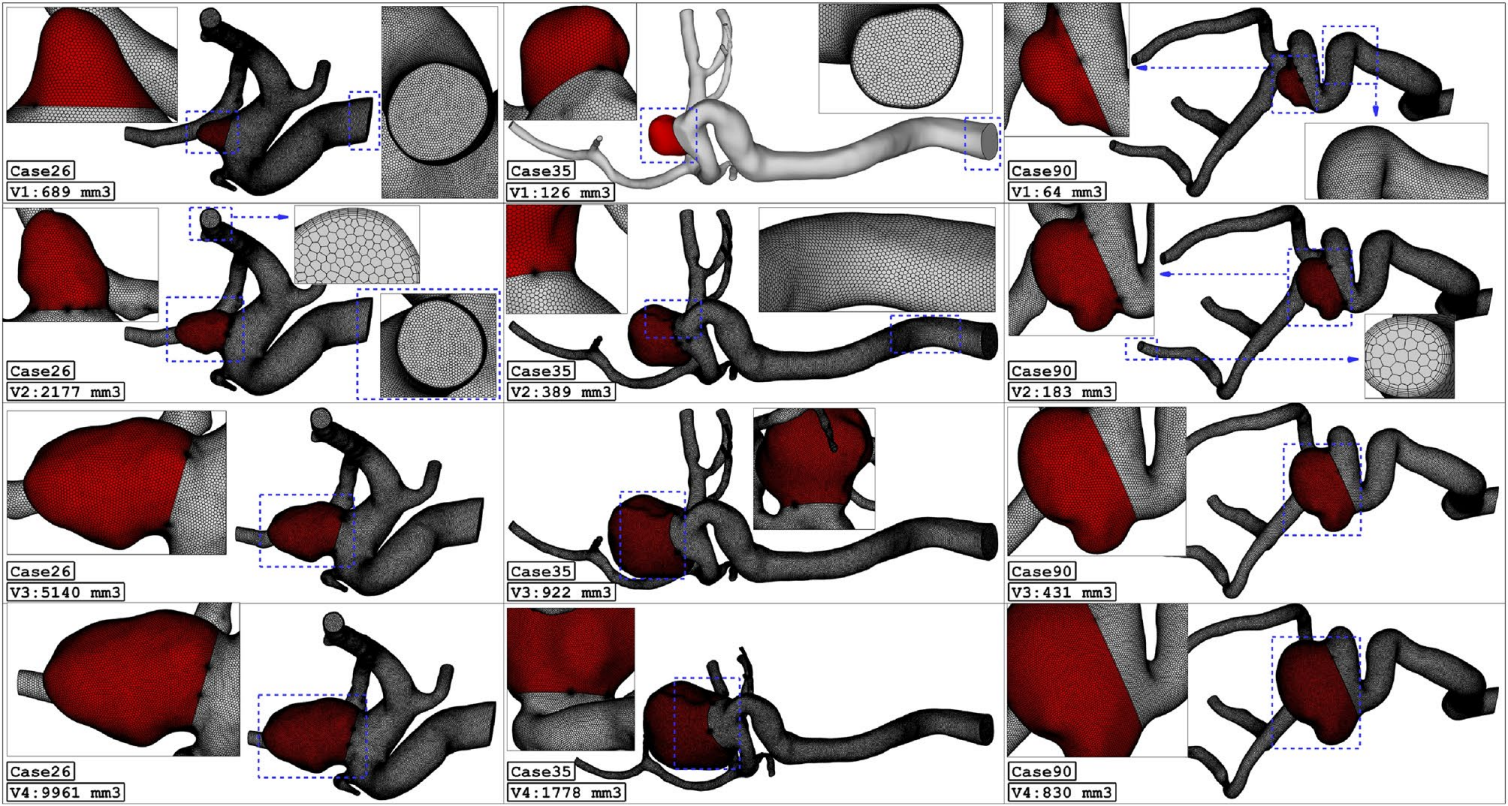
Grid generation for 3 different ICA cases.

The produced grid for the original and modified saccular aneurysms is displayed in Fig. 3. The resolution of the produced grid is almost homogeny inside the sac area while its intensity is higher near the sac wall due to the importance of this section. As expected, the number of produced grids is increased when the sac area is scaled up. The sac section area is colored red and the parent vessel is gray color.

## Results and discussion

The influences of the aneurysm evolution on the hemodynamic factors of selected aneurysm are presented in Table 2. In this table, the model number 3 is related to the original shape of the aneurysm which was introduced in Table 2.

**Table 2:** Obtained data from our simulations.

	Model Number	Aneurysm Volume (mm^3^)	WSS_Min (Pa)	WSS_mean (Pa)	OSI_mean	Wall pressure_mean (Pa)	Aneurysm Velocity_mean (m/s)
Case26	I	689	0.002065	0.075	0.014	15,870	0.0107
	II	2177	0.000081	0.035	0.010	15,871	0.0061
	III	5140	0.000355	0.026	0.006	15,868	0.0064
	IV	9961	0.000257	0.019	0.007	15,869	0.0057
Case35	I	126	0.627810	17.71	0.006	20,741	0.6086
	II	389	0.249632	9.53	0.014	21,241	0.3874
	III	922	0.052047	6.69	0.025	20,696	0.3032
	IV	1778	0.028644	4.10	0.056	21,325	0.1839
Case90	I	64	0.587990	15.99	0.040	19,396	0.5323
	II	183	0.582664	12.35	0.019	19,567	0.4372
	III	431	0.356871	8.14	0.013	19,346	0.3105
	IV	830	0.052492	5.25	0.040	19,650	0.2205

Among the various hemodynamic factors associated with aneurysm evolution and rupture risk, wall shear stress (WSS) is considered to have a critical change. Wall shear stress represents the frictional force exerted by blood flow on the inner surface of the blood vessels, including the aneurysm wall.

Studies have shown that alterations in WSS play a significant role in aneurysm growth, progression, and rupture. High wall shear stress has been associated with increased risk of aneurysm rupture, as it can lead to endothelial dysfunction, inflammation, and remodeling of the vessel wall. Prolonged exposure to elevated WSS can weaken the aneurysm wall, making it more prone to rupture.

Furthermore, regions of low or disturbed wall shear stress can also contribute to aneurysm formation and growth. Low WSS regions may promote the formation of thrombus or blood stagnation within the aneurysm, leading to further pathological changes and increasing the likelihood of rupture.

The distribution and magnitude of wall shear stress within the aneurysm are critical factors in assessing rupture risk. CFD simulations, as mentioned earlier, can estimate the WSS distribution within the aneurysm and help identify regions of elevated or altered WSS. These regions can serve as potential indicators of increased rupture risk or areas requiring closer monitoring.

It’s important to note that while WSS is a significant factor, the overall understanding of aneurysm evolution and rupture risk is multifactorial. Other hemodynamic factors, such as flow velocity, pressure, and oscillatory shear index, also contribute to the complex interplay involved in aneurysm development and rupture. Therefore, a comprehensive assessment that considers multiple factors is necessary for a more accurate evaluation of rupture risk in cerebral aneurysms.

**Figure 4:**
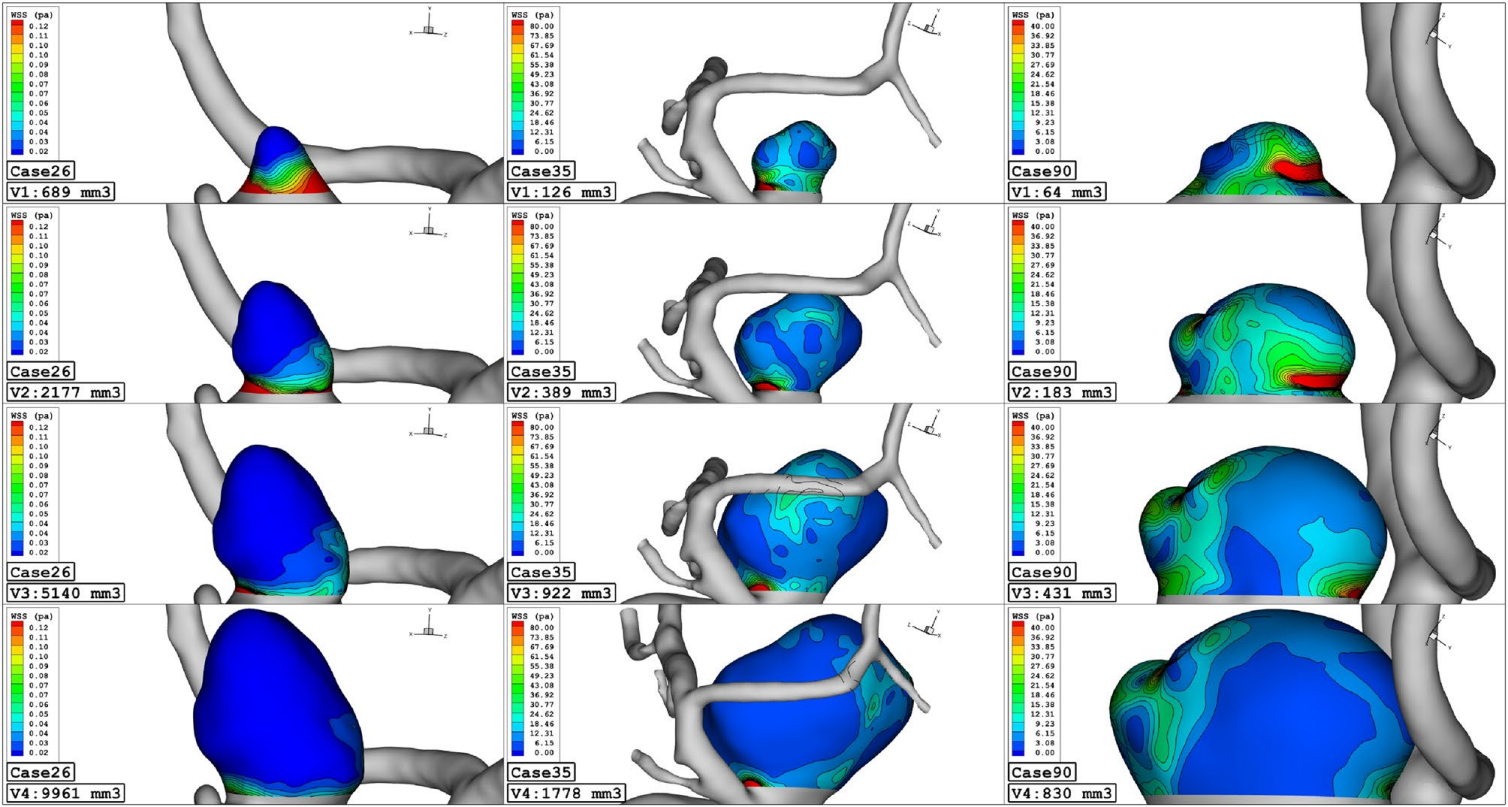
WSS contours (Peak systolic) in different sac volume.

**Figure 5:**
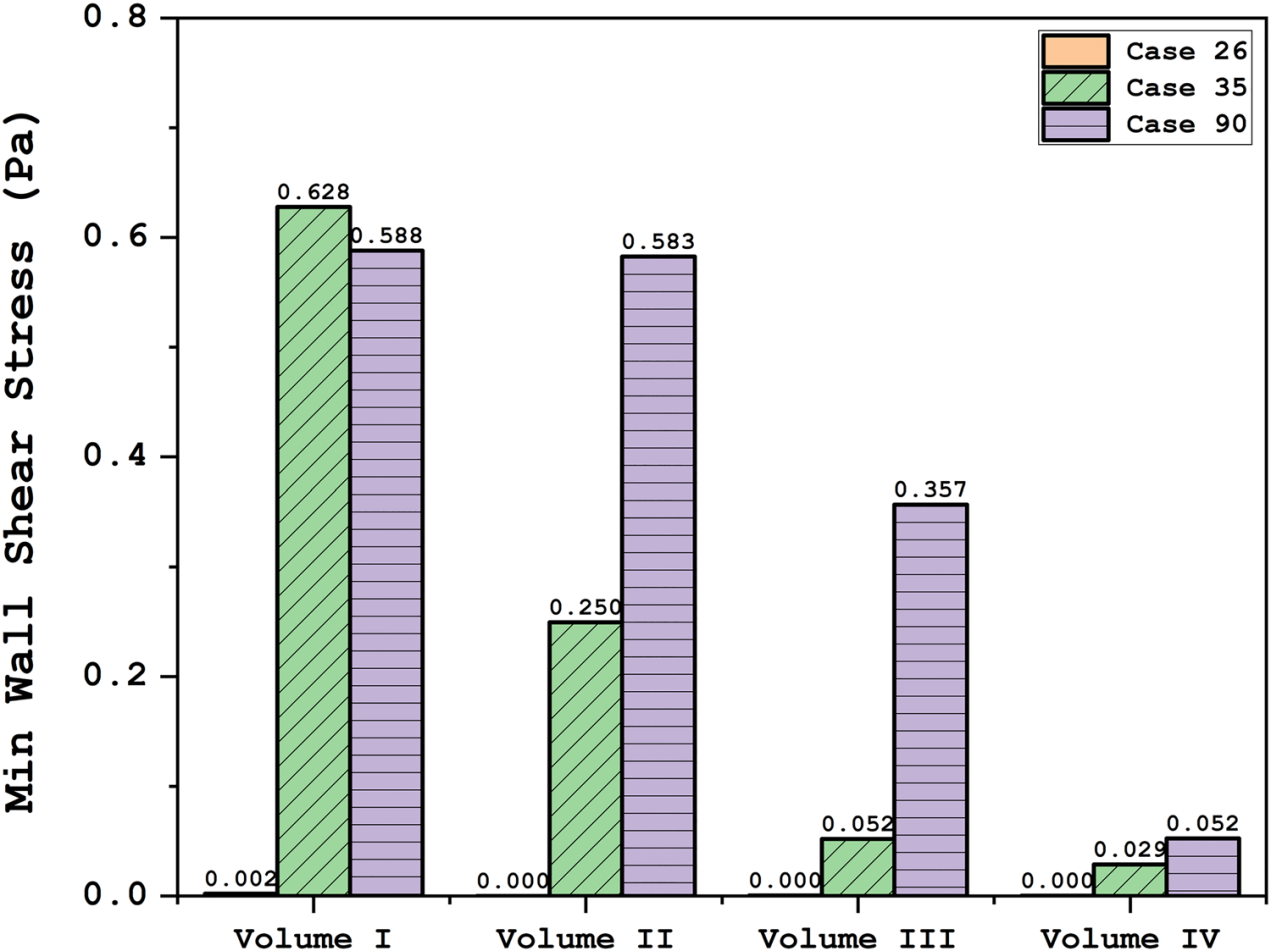
Minimum wall shear stress at peak systolic.

**Figure 6:**
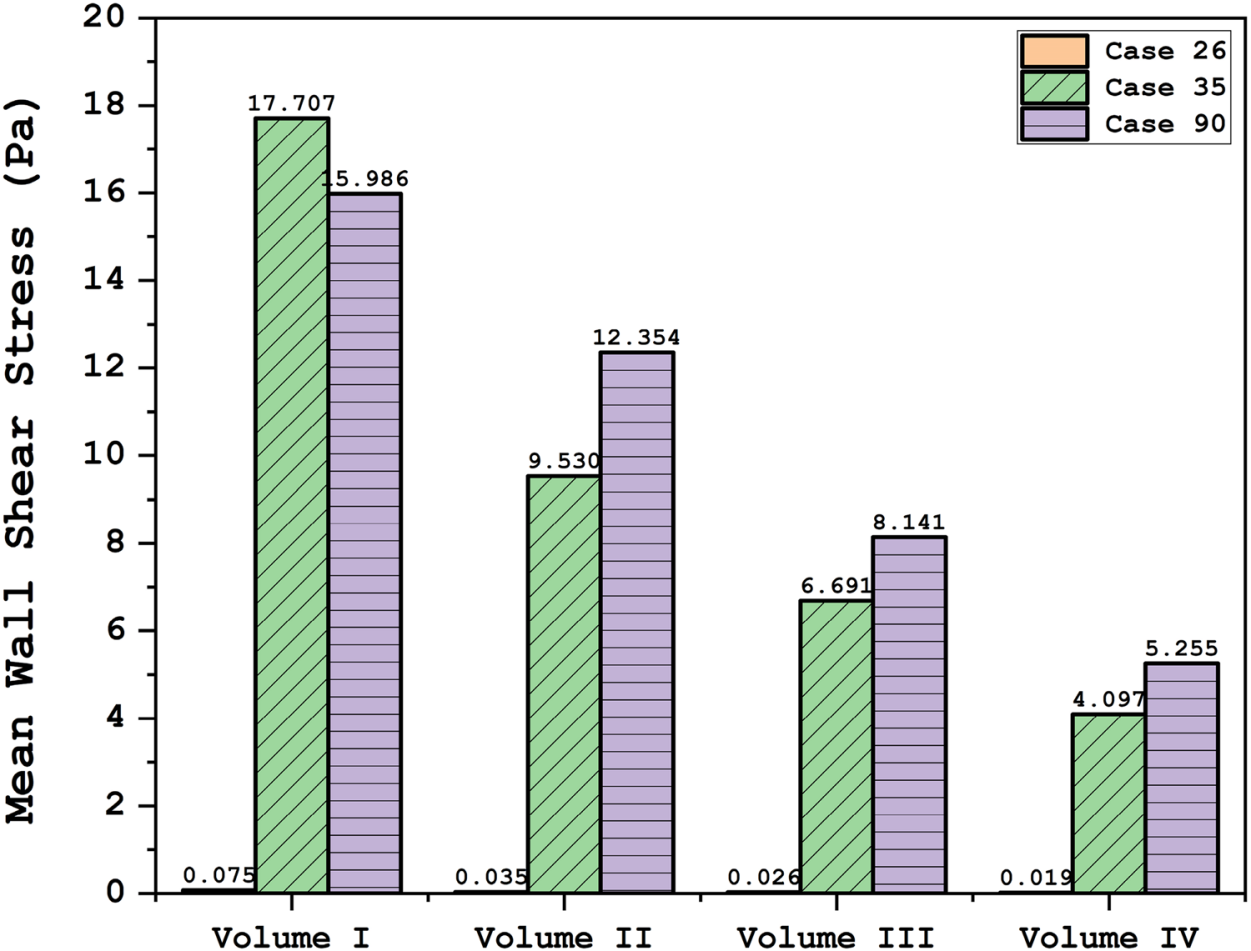
Mean wall shear stress at peak systolic.

To attain a reasonable comparison of these cases, these data are analyzed via plot and contour in the upcoming sections. Figure 4 illustrates the contour of the WSS on the sac surface in different scales of the ICA aneurysms. In case 26, the critical high WSS region occurs in the near neck section and this region expands when the volume of the aneurysm is reduced. In case 35, the critical region with high WSS happens on the sharp curvature of the sac region. The growth of the aneurysm also limited regions with high WSS. The evaluation of case 90 in different scales also shows that growth of the aneurysms decreases sharp curvature on the sac surface and consequently region with high WSS is limited. The variation of min and mean wall shear stress of the selected aneurysms in different scales of growth is also presented in Figs. 5 and 6, respectively. A comparison of minimum wall shear stress indicates that the factor drops meaningfully by expanding the aneurysms. The same pattern is also noticed in the mean WSS. The trend of the Mean WSS indicates that this factor changes more reasonably by the volume evolution.

**Figure 7:**
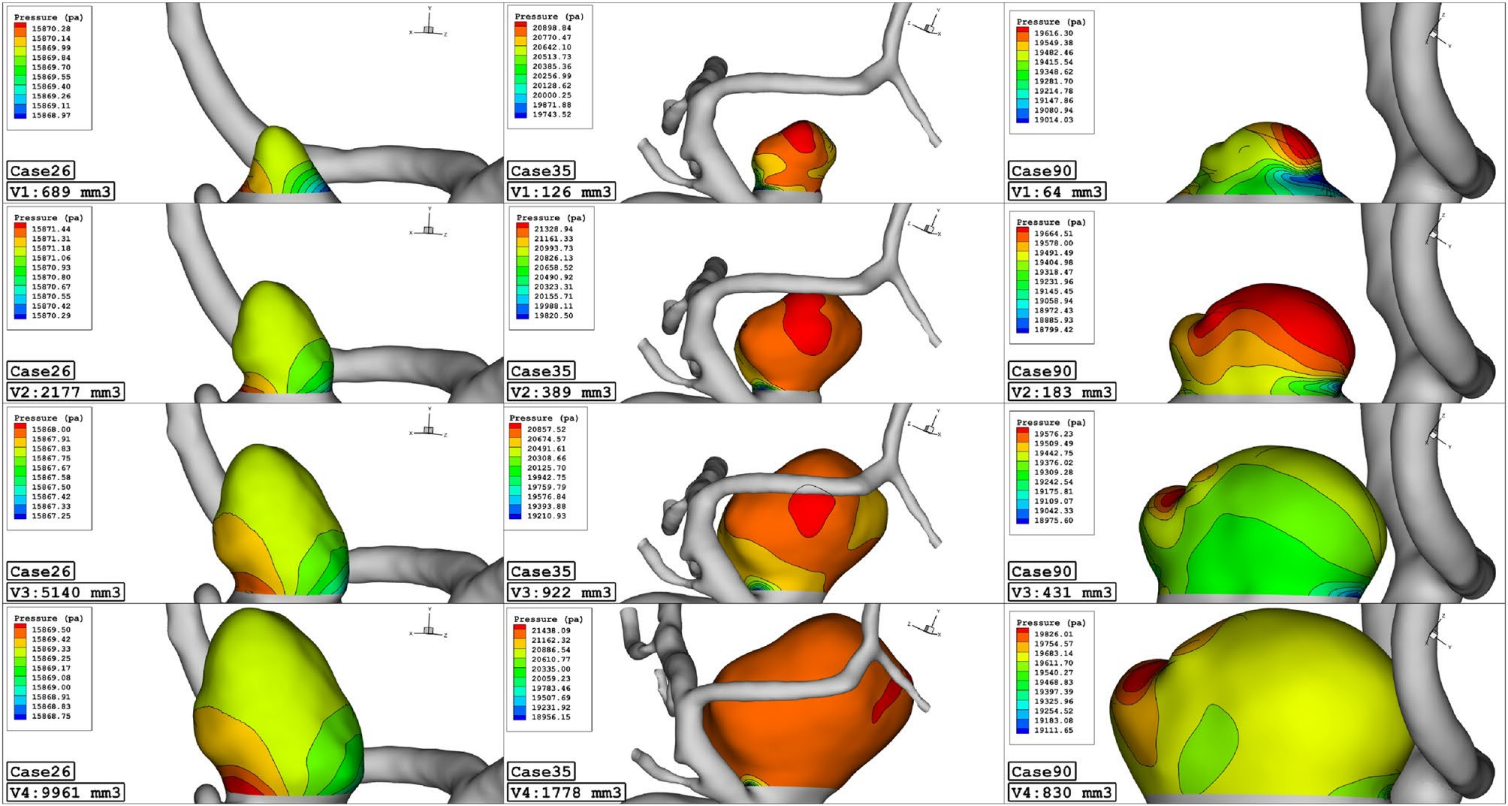
Wall pressure contours (Peak systolic) in different sac volume.

**Figure 8:**
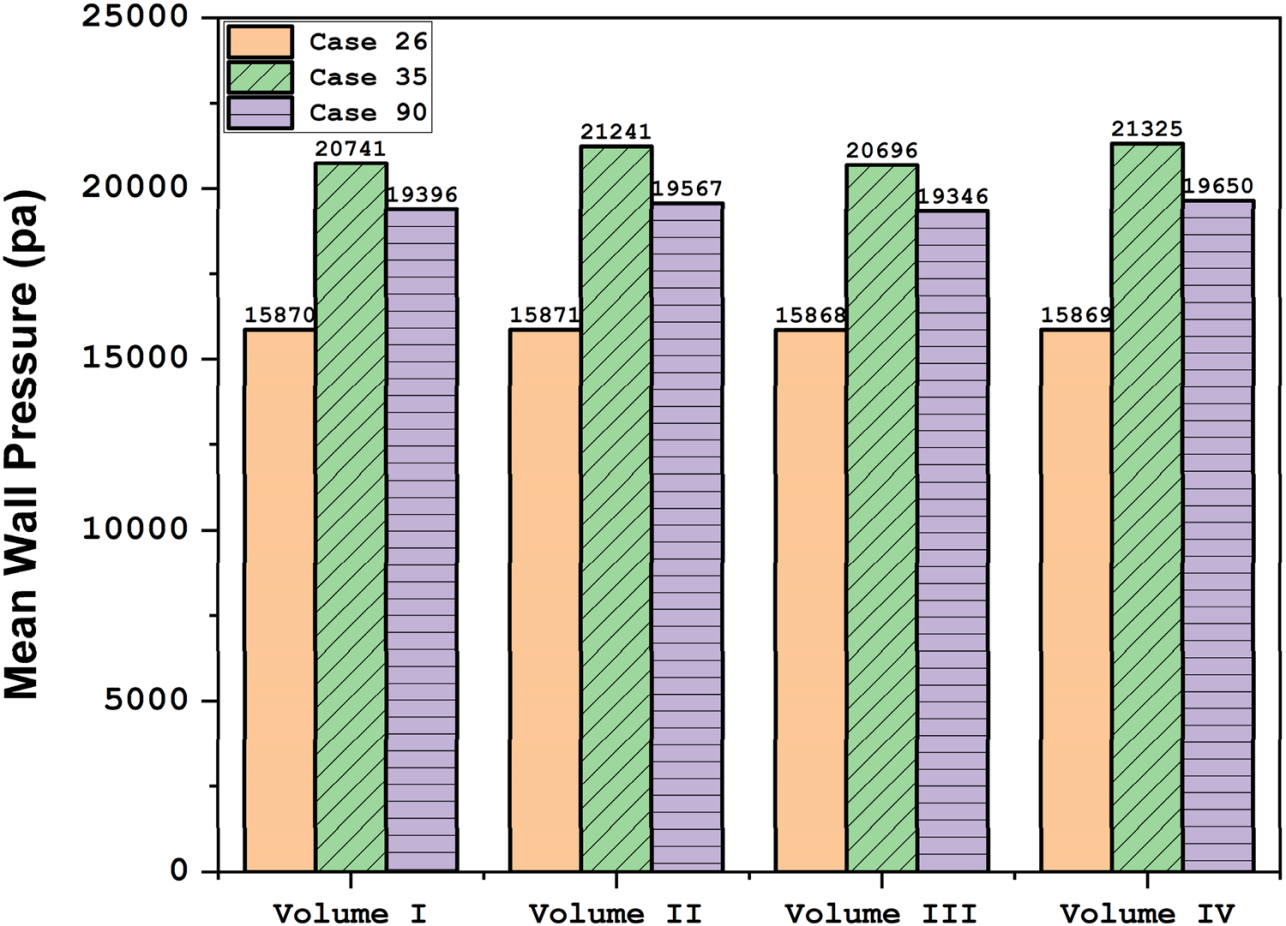
Mean wall pressure at peak systolic.

Figure 7 demonstrates the pressure change on the sac surface in different scales of aneurysm growth at stage of peak systolic. The change in the sac volume results in lower curvature on the sac surface and consequently, the maximum pressure zone restricted on the sac surface. Although the pressure contour illustrates a meaningful decrease on the aneurysm wall, the mean pressure of the selected aneurysm does not change in the evolution process as presented in Fig. 8.

**Figure 9:**
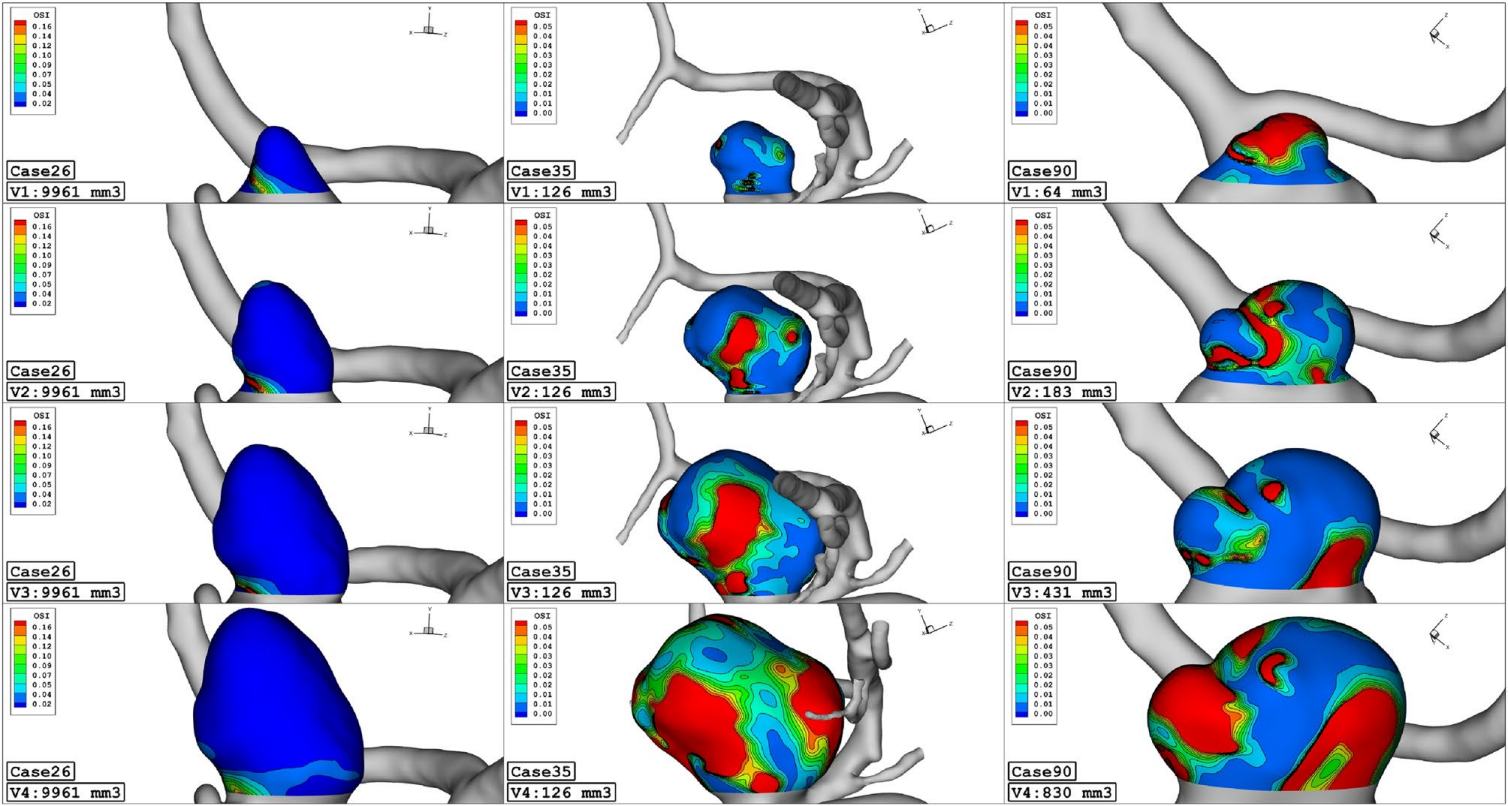
OSI contours (Early diastolic) in different sac volume.

**Figure 10:**
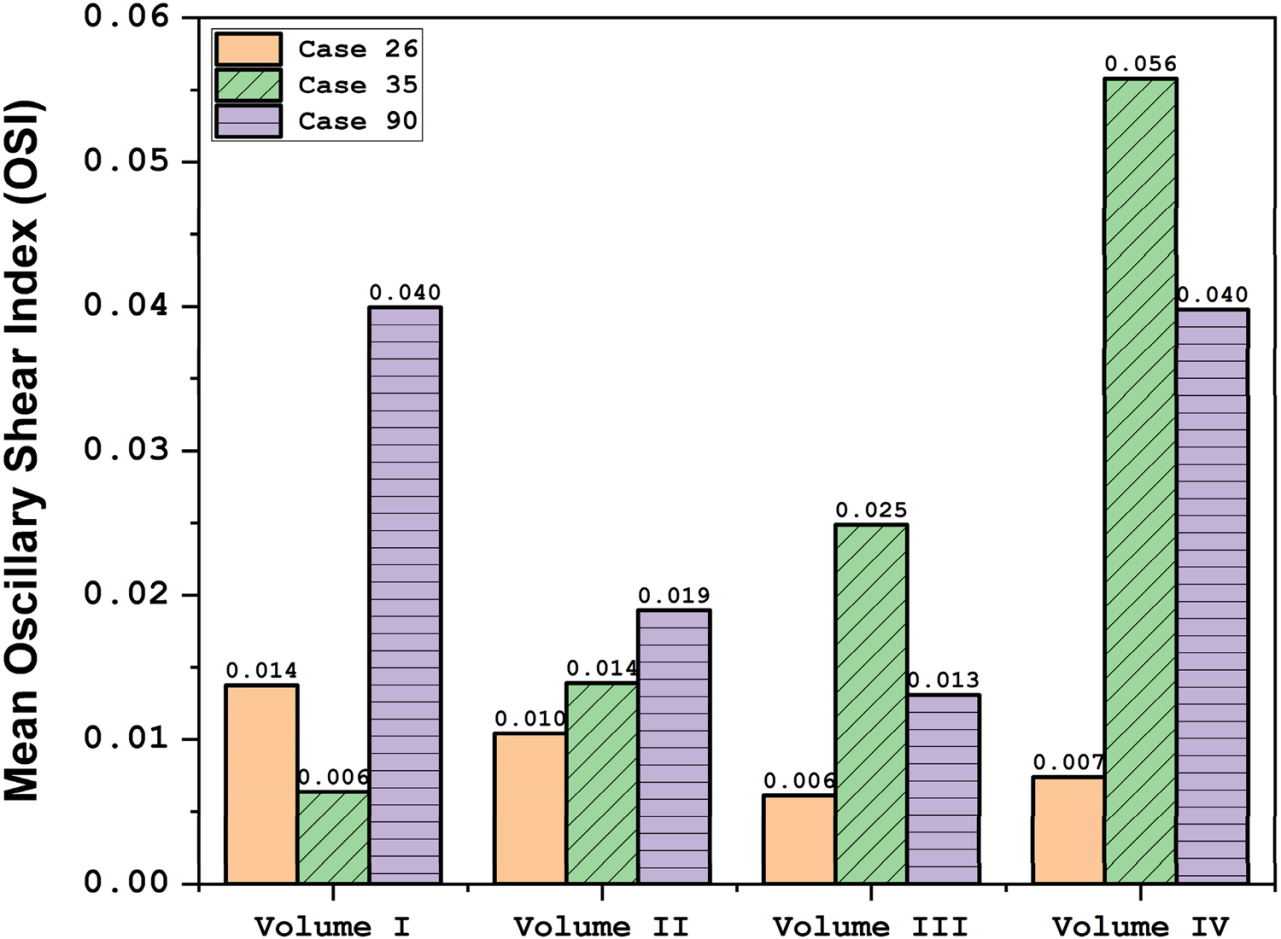
Mean OSI at early diastolic.

OSI contour at early diastolic of selected saccular aneurysms is displayed in Fig. 9. In case 26, the evolution of the aneurysm does not change OSI on sac volume. In both case35 and case90, the evolution of the aneurysm results in a larger critical OSI region on the sac surface. The oscillatory index represents the region where rupture could happen. The value of OSI shows that OSI is extended by the expansion of the aneurysm volume in the evolution process. The archived quantitative data (Fig. 10) confirms that the evolution of the aneurysm by scaling up the size results in a higher mean OSI value (more than 100 growth) on the sac surface (especially in case 35 and case 90). Hence, the aneurysm rupture increases in these cases when the evolution happens.

**Figure 11:**
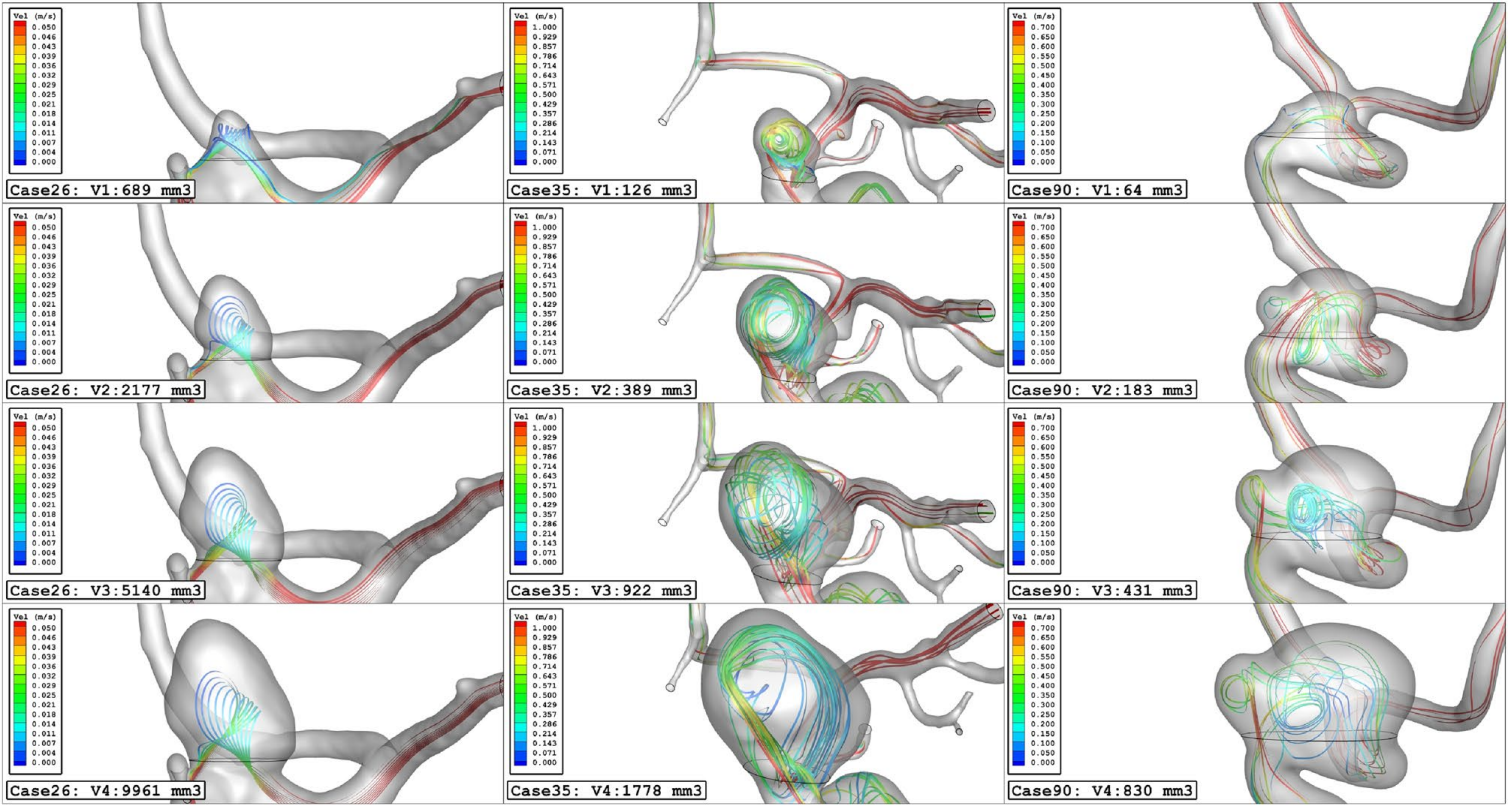
Streamlines (velocity at peak systolic) in different sac volume.

**Figure 12:**
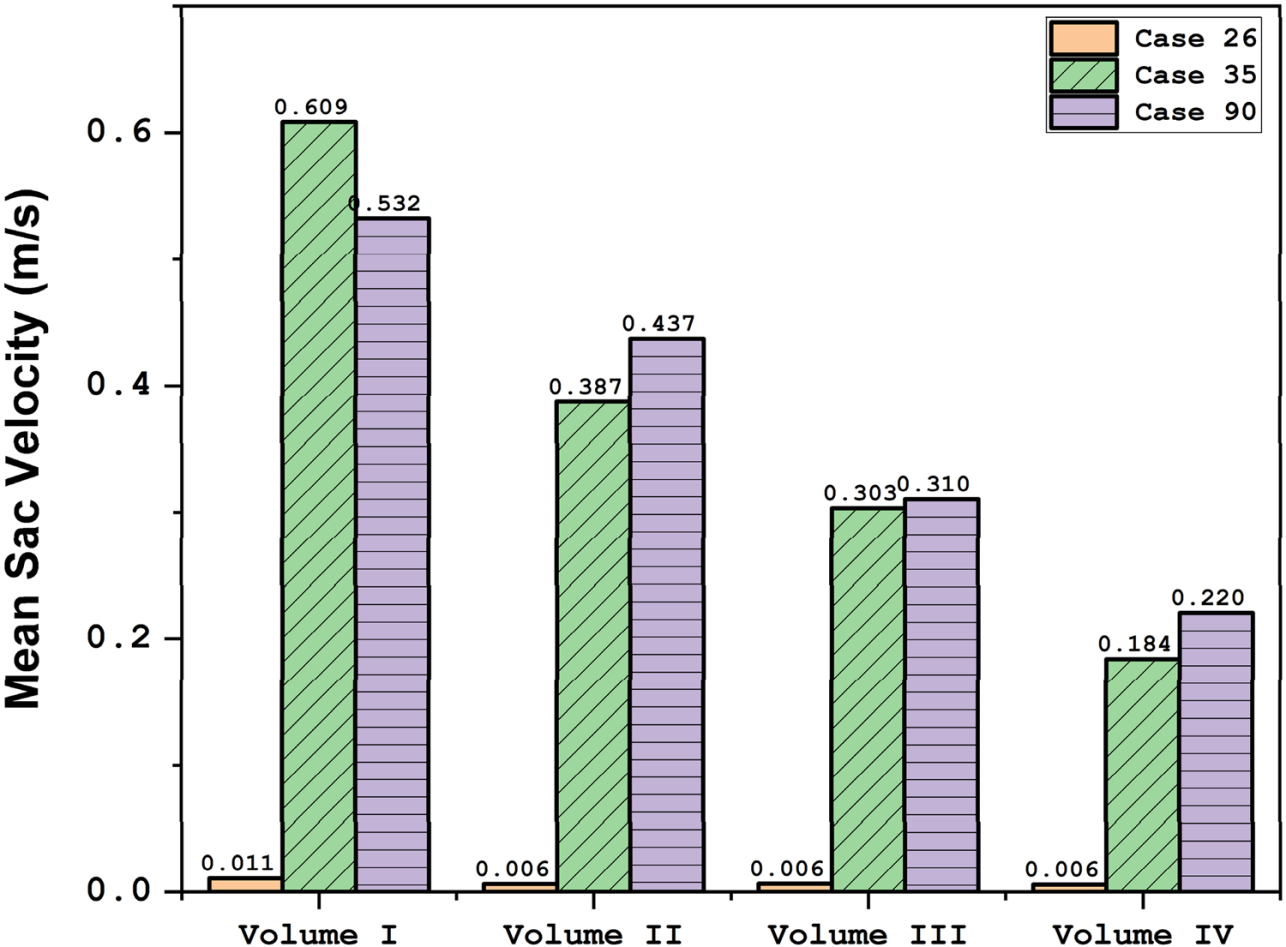
Mean sac velocity at peak systolic.

**Figure 13:**
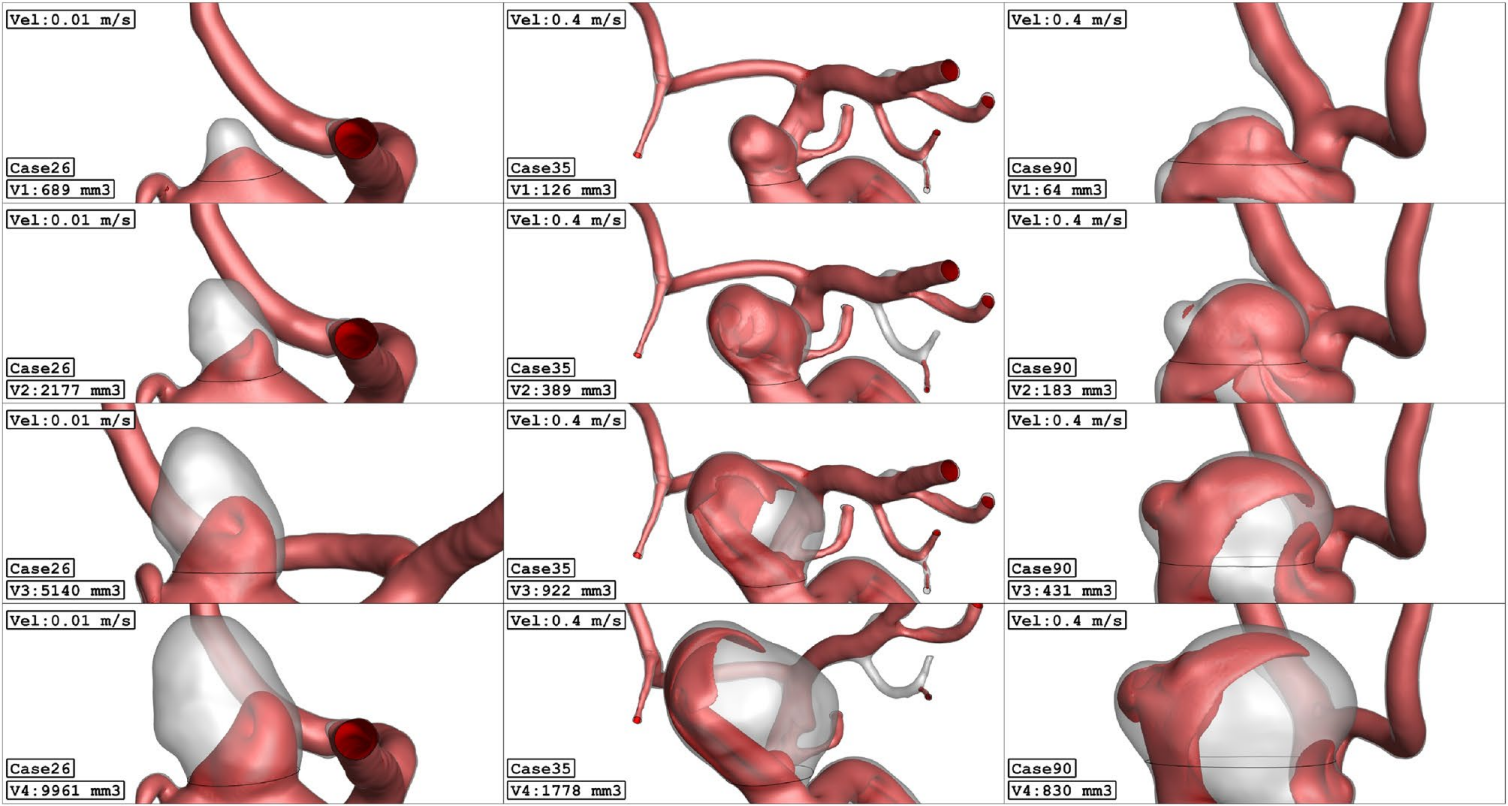
Iso-Surface (velocity at peak systolic) in different sac volume.

Due to the importance of the blood stream velocity, Fig. 11 illustrates the blood stream in these aneurysm cases in the evolution process. As demonstrated in the figure, the expansion of the aneurysm size results in the lower velocity of blood stream in the sac section region. The value of the mean sac velocity of the selected aneurysms (Fig. 12) in the evolution process also verifies these findings. To observe the blood flow structure, Fig. 13 illustrates the iso-velocity of the blood flow in the sac region. Based on these images, the main portion of the iso-velocity happens near the aneurysm vessel and the evolution of the aneurysm limited high-velocity blood flow in this area.

## Conclusion

The present article offers significant results about the change of hemodynamic factors in the evolution process of ICA aneurysm. This study visualizes the blood stream inside the three different saccular ICA aneurysms in scale-up and down versions. The structure of the blood stream in the evolution process is compared to disclose the risk of aneurysm rupture in the process of aneurysm evolution. The contour of WSS shows that the expansion of the aneurysm size reduces this factor on the sac wall. Besides, the mean velocity of the blood decreases when the aneurysm size is scaled up. Moreover, the pressure on the sac wall decreases as the volume of the sac is increased. However, the mean OSI value increases when the aneurysm volume is enlarged.

## Data Availability

All data generated or analysed during this study are included in this published article.
